# Psychological fortitude model for digitally mindset working adults

**DOI:** 10.3389/fpsyg.2022.985749

**Published:** 2022-11-24

**Authors:** Ingrid Potgieter, Nadia Ferreira

**Affiliations:** Department of Human Resource Management, University of South Africa, Pretoria, South Africa

**Keywords:** agile adaptable attributes, career adaptability, career wellbeing, digital age, psychological fortitude, remote working, value-orientated psychological contract

## Abstract

**Introduction:**

The inception of Industry 4.0 (which includes smart digital technologies and intelligence), as well as the rapidly enforced adoption of the technological revolution due to the lockdown regulations during the COVID-19 pandemic, brought new situational demands, challenges and opportunities for both employees and organizations across the globe. Individuals are required to develop personal enablers (both intrapersonal and intradigital attributes) to optimize their psychological fortitude. Research on the intrapersonal resources needed by employees to have the fortitude to adapt to remote working conditions as a result of the digital era, is currently lacking. The igital era brought about the question of how individuals’ career adaptability and career wellbeing (as a set of agile adaptable attributes) relate to their perceptions of the value-oriented psychological contract, and whether these intrapersonal resources can contribute to a psychological fortitude model for remote working employees.

**Method:**

This study utilized a survey method to investigate the correlations between agile adaptable attributes and the valueoriented psychological contract of global digital-mindset human resource and financial service organizations. Based on further canonical correlations, structural equation modeling was conducted to develop and recommend a psychological fortitude model for remote working adults in the digital age.

**Results:**

Close theoretical and empirical associations were found between career adaptability and career wellbeing (as agile adaptable variables) and the perceived value-orientated psychological contract.

**Discussion:**

This study proposed a psychological fortitude model (consisting of intrapersonal resources) that organizations and career practitioners can use as a basis to enhance employees’ psychological fortitude in the digital age, as well as for further career research.

## Introduction

With the inception of the Fourth Industrial Revolution, increased emphasis was placed on the inquiry into positive human functioning in the fast-changing, digital workspace (Brown et al., 2017). Industry 4.0, together with the rapid changes brought about by the COVID-19 pandemic, steadily created the new normal working context, characterized by technology, remote working and social distancing (Potgieter, 2022).

Research studies by Brown et al. (2017) pointed out that a positive psychological state is dependent on the situational context (such as the career space context as emanating from the digital era and remote working conditions). Individuals’ perceptions, interpretations and cognitive appraisal of their situational demands and the resources and support available to positively cope and adapt to the challenges, changes and stressors of the work context and conditions, as well as their ability to self-manage and adapt, are significant enablers of positive human functioning (Coetzee, 2019). According to Pretorius and Padmanabhanunni (2021), sufficient adaptive cognitive appraisals about oneself, one’s family, social support systems, organizational support and the immediate external environment results in fortitude. Fortitude is the psychological strength to cope amidst adversity and to maintain wellbeing (Pretorius and Padmanabhanunni, 2021). Pretorius and Padmanabhanunni (2021) also describe fortitude as a protector of personal and psychological wellbeing. Fletcher and Sakar (2016) pointed out that fortitude is different than resilience, as fortitude relate to the psychological strength to find courage during challenging times whereas resilience relate to the psychological ability to recover from misfortune or difficult times.

Psychological fortitude relates to the intrapersonal strength of an individual to face difficult situations and uncertainty, such as the challenges and obstacles posed by the digital era and the new demands and challenges of remote working. Psychological fortitude is therefore the cognitive endurance of employees to survive, thrive and cope in the digital era. An overarching premise is that psychological fortitude is a psychological state of personal development and success in uncertain, unstable and stressful organizational contexts. According to Coetzee (2019), employees who showcase positive psychological states experience higher levels of wellbeing, greater job satisfaction and engagement, and a perceived high level of job performance.

The inception of Industry 4.0 (which includes artificial intelligence and smart digital technologies), as well as the rapidly enforced adoption of the technological revolution due to the recent worldwide lockdown regulations during the COVID-19 pandemic, brought new challenges, requirements, demands and opportunities for both employees and organizations. Individuals should develop personal enablers (both intrapersonal and intradigital attributes) to optimize their psychological fortitude. Research on the intrapersonal resources needed by employees to have the fortitude to not only succeed, but to thrive in the digital era, is currently lacking.

The digital era, characterized by turbulent working conditions, the gig economy, remote working, fast changing technological innovation and globalization, contributed to the birth of the value-oriented psychological contract of the career-agile employee (Scheel and Mohr, 2013; Ghislieri et al., 2018; Coetzee, 2021; Veldsman, 2021). According to Coetzee (2021), value-oriented content refers to the expectation of an employee that the obligated organizational contributions agreed upon will meet their own personal career values and needs, will contribute to a higher and valuable organizational objective, and that the employer will provide the agreed upon value-oriented organizational support. Aderibigbe (2021) noted that the unwritten agreement between employer and employee relates to the individual’s need for career meaningfulness, which will provide them with the ability to progress and evolve in their career identity and to make significant contributions or meet the expectations/requirements of the customers they serve, the larger community, as well as the environment. Singh et al. (2020) suggested that the rapid changes in the digital era required a new way of understanding the employment relationship. In addition, the workspace now also includes a younger generation (the digital natives) that has its own set of perceptions, cognitive appraisals and expectations regarding the employment relationship (Deas, 2021). Karani et al. (2021) emphasized the need for research on the nature of the psychological contract and the contributors to the employment relationship as the foundation of the psychological contract. Coetzee (2021) also reiterated that the digital era (characterized by digital relationships and communication channels) required a new understanding and innovative methods of managing the psychological contract (Table 1).

**Table 1:** Value-oriented psychological contract dimensions.

Employee’s primary and secondary obligated inputs	The *employee’s primary obligated inputs* encompass the responsibility or obligation of the employee to achieve job requirements, to provide inputs, ideas and efforts in the execution of job roles, to act ethically and honestly and to positively participate and implement creative thinking in the execution of expected tasks. The *employee’s secondary obligated inputs* refer to the responsibility to work hard, be committed to task completion as well as the organization and its brand, to accomplish the organization’s objectives, values, vision and mission and to consciously contribute positively to the organization’s performance, objectives, culture and success.	Aderibigbe (2021); Deas (2021); Deas and Coetzee (2021)
Organization’s obligated outcomes	The *organization’s obligated outcomes* encompass the employee’s expectations regarding what they should receive in exchange for their inputs (for example, career development support, fair and respectful treatment, work-life balance, fair remuneration and benefit packages, and challenging and meaningful work).	Rousseau (2001, 2008); De Vos et al. (2003); Isaksson et al. (2003); Coyle-Shapiro and Conway (2005); Freese et al. (2008); Schwieger and Ladwig (2018); Deas and Coetzee (2021).
Employee’s obligated inputs delivered	The employee’s obligated inputs delivered include an equity check point that contributes towards the perceived fit between the person and the environment (or misfit). A perceived misfit may result in either a renegotiation of the employment relationship or a resignation to search for a better fit elsewhere.	Darrow and Behrend (2017); Jiang (2017); Deas and Coetzee (2021); Ferreira et al. (2022); Guan et al. (2021).
Employer’s obligated psychological contract fulfilment	The employer’s obligated psychological contract fulfilment also entails an equity checkpoint towards the fulfilment of the expected or obligated employment relationship. A perceived misfit may result in renegotiation of the person-environment fit, or in searching for a better fit elsewhere.	Luthans (2011); Darrow and Behrend (2017); Jiang (2017); Deas and Coetzee (2021); Ferreira et al. (2022); Guan et al. (2021).

Agile adaptable employees have the ability to adapt and effectively carry out tasks amidst change, technology and uncertainty. Potgieter (2021) found career adaptability to be an essential agile adaptable dimension within the digital career space. Coetzee (2021) in turn found that career wellbeing should be considered as an essential agile adaptable dimension required for effective coping in the new normal digital world of work. The digital era raised the question of how individuals’ career adaptability and career wellbeing (as a set of agile adaptable attributes) relate to their perceptions of the value-oriented psychological contract and if these intrapersonal resources can contribute to a psychological fortitude model for employees in the digital era. The general aim of this study was thus to propose a psychological fortitude model (consisting of intrapersonal resources) that organizations and career practitioners can use as a basis to enhance employees’ psychological fortitude in the digital age.

## Literature review

### Psychological contract

The value-oriented psychological contract is based on the principles of the equity theory of Adams (1963). Deas (2021) summarized these principles as the motivation of employees by their intra-motivation to maintain a balance between, on the one side, their own agreed upon attitudinal and performance-orientated inputs and efforts that they contribute within their jobs and tasks, and on the other hand, in exchange for their input and effort, the agreed upon outcomes from the organization. This perceived input-outcome balance incorporates the assessment of the equity and fairness ratio between the psychological career value requirements (that is, the agreed upon inputs delivered) and the expected agreed upon organizational outcomes (Deas and Coetzee, 2021; Guan et al., 2021). Bal and Vink (2011) found that if the perception is present that a fair input-outcome is being achieved, and if employees believe that there is a balance between their personal goals, motives and values (Payne et al., 2008), a positive psychological contract appraisal, high job satisfaction and affective organizational commitment will be reached. Coetzee (2021) found that a positive perception regarding the value-oriented psychological contract results in creative thinking, enhanced organizational performance, enhanced employee performance, positive attitudes, positive participation in group tasks and teamwork, enhanced organizational commitment and loyalty towards the brand and mission of the organization. Veldsman (2021) confirmed that the contemporary psychological contract relies on a brief, short-term, equitable and transactional exchange of obligated or agreed upon employee-employer value matching. Deas (2021) developed four psychological-contract-orientated dimensions, which include (1) *“employee primary and secondary obligated inputs*,” (2) *“organisational obligated outcomes,”* (3*) “employee obligated inputs delivered*,” and (4) *“employer obligated psychological contract fulfilment.”*

No research has been found to date on the contributors to the value-oriented psychological contract, specifically in the digital new-normal career space (which entails, to a large degree, employees working remotely).

Numerous studies were found explaining the correlation between the psychological contract and organizational commitment (McDonald and Makin, 2000; Lub et al., 2012), as well as between career adaptability and organizational commitment (Ferreira and Coetzee, 2013; Coetzee et al., 2017; Jabaar, 2017; Ferreira, 2019). However, to date, no research has been found on the influence of career adaptability on the value-oriented psychological contract.

### Career adaptability

The new world of work and fast changing digital era is characterized by frequent change and transitions between jobs, organizations and careers, which necessitate more agile and flexible adaptation on the part of employees (Rudolph et al., 2017). The ability to adapt and demonstrate that one can adjust is essential to effectively deal with the digital era’s extraordinary social, economic and technological changes that are reshaping the world of work (Johnston, 2018; Lent, 2018). Martin et al. (2019, p. 566) define career adaptability as “the skill to constructively regulate psycho-behavioral functions in response to new, changing, and/or uncertain circumstances, conditions and situations.” Career adaptability is defined as intrapersonal psychological capacities, functions and resources during the career management process. According to Hirschi (2018), these psychological capacities facilitate proactive adaption and successful alignment with the fast-changing digital world of work (Table 2).

**Table 2:** Dimensions of career adaptability.

Career concern	Career-related cognitive anticipation and preparation to respond to the demands, challenges and changes of the future job requirement and work environment.	Savickas and Porfeli (2012); Savickas (2013); Rudolph et al. (2017); Ginevra et al. (2018); Kirdok and Bolukbaşi (2018)
Career control	The degree of accountability that an individual accepts for their career future. Career control also includes the adoption of self-regulation strategies to adjust to the requirements of various settings.	Savickas and Porfeli (2012); Savickas (2013); Oncel (2014); Coetzee and Stoltz (2015); Rudolph et al. (2017); Ginevra et al. (2018)
Career curiosity	Curiosity is the intrinsic motivation to explore possible future selves and associated career possibilities and options.	Savickas and Porfeli (2012); Savickas (2013); Rudolph et al. (2017); Ginevra et al. (2018)
Career confidence	Alludes to the belief and having confidence in one’s own capability to achieve career goals amidst uncertain and unstable career conditions.	Savickas and Porfeli (2012); Savickas (2013); Rudolph et al. (2017); Ginevra et al. (2018)

Individuals with high levels of career adaptability experience more positive emotional dispositions (Johnston, 2018), can adapt to technological innovation and positively participate in agile learning and career navigation (Potgieter et al., 2021). Career adaptability connects the individual’s willingness and ability to adapt to changing career situations, such as the fast-changing digital requirements in the world of work (Hirschi et al., 2015).

From the literature, it is assumed that career adaptability will theoretically contribute to the value-orientated psychological contract.

Numerous studies were found on the correlation between wellbeing and the psychological contract (Bester, 2019; Duran et al., 2019; Collins and Beauregard, 2020), as well as wellbeing and employability (Coetzee and Engelbrecht, 2019), job satisfaction (Engelbrecht, 2019), organizational commitment (McInerney et al., 2015; Ferreira, 2019) and adaptability (Ferreira, 2019). However, to date, no research has been found on the influence of career wellbeing on the value-oriented psychological contract in the digital era.

### Career wellbeing

The digital era also significantly influenced the career wellbeing of employees (Loveder, 2017; Potgieter, 2019). Career wellbeing is an intrapersonal positive psychological capacity that reflects an employee’s long-term satisfaction with the outcomes, achievements, success and changes of their career within the challenges, rapid changes and complexities of the working context (Bester et al., 2019).

According to Coetzee et al. (2021), career wellbeing is a multidimensional construct which includes the facets of positive career affect, career networking/social support and career meaningfulness. Table 3 defines the facets of career wellbeing.

**Table 3:** Facets of career wellbeing.

Positive career affect	Relates to positive emotions consequent to psychological states. Individuals with positive career affect mostly feel satisfied with the given conditions to achieve their career goals.	Tugade et al. (2004); Engelbrecht (2019); Potgieter et al. (2021)
Career networking / social support	Refers to the perceptions of an individual that they have a network of people who support their career goals and that this support network can easily be approached to assist in achieving their career goals. Individuals with a sound career network / social support believe that feedback from the social support network may enhance their strengths.	Reich et al. (2010); Potgieter (2019); Ferreira (2021); Potgieter (2021); Potgieter et al. (2021).
Career meaningfulness	Alludes to the belief that one’s career has meaning and that being involved in this career is a matter of personal choice. Individuals who experience career meaningfulness, see their career as worthwhile and valuable and believe that their careers contribute to the bigger picture and enhance lives. Believing that one’s career has meaning creates optimism about the future and provides motivation to cope with stressful working conditions.	Allan et al. (2020); Coetzee et al. (2021), Potgieter et al. (2021)

From the literature, it is assumed that wellbeing will theoretically contribute to the value-orientated psychological contract.

## Materials and methods

### Participants

The sample in this study (*N* = 293) consisted of national and international digital-oriented financial service and human resource management organizations. These organizations were predominantly located in South Africa (70%), while the rest of the sample organizations were based in Zimbabwe (15%) and in Europe (15%). The sample was almost equally represented by gender (men 54% and woman 46%). The majority of the sample belonged to the Black race groups (African/Indian/Asian/Colored: 63%), while 37% of the participants belonged to the white race groups.

### Instruments

*The career adaptability scale* (Savickas and Porfeli, 2012), a 23-item scale, was used to measure the dimensions of career adaptability, which includes *career concern* (6 items, e.g., “Realizing that today’s choices shape my future”); *career control* (6 items, e.g., “Making decisions by myself”); *career curiosity* (6 items, e.g., “Exploring my surroundings”), and *career confidence* (5 items, e.g., “Solving problems”). The respondents had to rate each item on a seven-point Likert-type scale, where 1 represented “strongly disagree” and 7 represented “strongly agree.” Several previous studies have confirmed the construct validity of this instrument (Savickas and Porfeli, 2012). Ndlovu and Ferreira (2019) reported internal consistency reliabilities ranging from 0.57 (concern) to 0.87 (overall adaptability).

The *career well-being scale* (Coetzee et al., 2021), a 14-item scale, was used to measure the three facets of career well-being. The scale measures three states of career well-being: the *affective career state* (6 items, e.g., “I feel supported in achieving my career goals”); the *career networking/social support state* (4 items, e.g., “I have a feedback community that helps me stay in touch with my personal strengths and areas for enrichment”); and the *state of career meaningfulness* (4 items, e.g., “My career is interesting and makes me excited”). The items were rated on a seven-point Likert-type scale, where 1 represented “strongly disagree” and 7 represented “strongly agree.” Initial research reported construct validity, as well as acceptable internal consistency reliability of the career well-being scale (Coetzee et al., 2021). The Cronbach alphas obtained for the subscales were 0.86 (affective career state), 0.85 (career networking/social support state) and 0.87 (career meaningfulness).

The *psychological contract inputs-outcomes inventory* (PCIOI; Deas and Coetzee, 2021), a multi-level 46-item scale, measures 4 dimensions of employees’ value-oriented psychological contract perceptions. The dimension of *employee inputs* measures perceptions about primary job performance responsibilities and secondary attitudinal responsibilities (12 items, e.g., “I feel obligated to meet performance requirements” and “I feel obligated to fulfil the organization’s vision, mission and its brand”). The dimension of *organizational outcomes* (29 items) measures the expectation employees have of their organization. This expectation includes facets of culture (e.g., “I expect clear goals and job role expectations*”)*, career development opportunities (e.g., “I expect to receive learning/coaching/mentoring on the job”), rewards (e.g., “I expect a fair compensation structure”), relationships (e.g., “I expect mutual respect between colleagues”), work-life balance (e.g., “I expect the flexibility in terms of where and when I do my job”), and job characteristics (e.g., “I expect innovative work challenges”).

The third dimension of the PCIOI measures the perception that the employee has regarding the fulfilment of the psychological contract, in delivering on expectations. This dimension is measured in 5 items (e.g., “I feel as a whole the organization has fulfilled my expectations”). The final dimension is a self-reflective checkpoint for employees, where they reflect on whether they feel that they have *delivered on their primary and input obligations* toward the organization (2 items, e.g., “I feel I delivered on the secondary employee inputs”). The items were rated on a 5-point Likert-type scale (1 = not at all; 5 = to a great extent). Construct validity and the internal consistency reliability of the scale were confirmed by Deas and Coetzee (2021).

### Procedure

The professional social media platform LinkedIn was used to gather the data. The message functionality of LinkedIn was used to send out a hyperlink which contained the survey to the researcher’s professional network on LinkedIn. Participants were invited to voluntarily participate and anonymously complete an online survey *via* the electronic link. A total of *N* = 293 respondents provided informed consent and participated in the study. No missing values were found in the data set and the data were analyzed using the SPSS (Version 27) statistical program.

### Ethical considerations

Ethical clearance to conduct the research was obtained from the University of South Africa (ERC Ref#: 2020_CEMS/IOP_014).

### Data analysis

Bivariate correlation analysis was performed to determine the existence of associations between the career adaptability, career wellbeing and value-oriented psychological contract dimensions. Canonical correlation analysis was performed to determine the strength of the overall variance shared between agile adaptable canonical variate (career wellbeing and career adaptability) as the independent variable and value-orientated psychological contract dimension (dependent variable). The structural model fit between the agile adaptable canonical variate and the value-orientated psychological contract dimensions was measured using SEM (structural equation modeling) with the maximum-likelihood (ML) estimation method. The root mean square error of approximation (RMSEA), chi-square test and the standardized mean square residual (SRMR) were used to assess the goodness-of-fit statistics.

The comparative fit index (CFI) and Tucker–Lewis index (TLI) as goodness-of-fit indices were also used to evaluate the model fit. When the CFI and TLI values are equal to or higher than 0.9, the RMSEA is equal to or lower than 0.08 and the SRMR is equal to or lower than 0.05, the model can usually be accepted as a good fit (Garson, 2008).

## Results

### Descriptive statistics and correlations

Table 4 reports the descriptive statistics obtained (means, standard deviations, internal consistency, reliabilities), as well as the correlations between the study variables. The career wellbeing variables had a positive and significant relationship with the career adaptability variables (*r* ≥ 0.24 ≤ 0.70; small to large practical effect; *p* ≤ 0.001). Except for the organizational outcomes of career development and rewards, the career wellbeing variables significantly and positively correlated with most of the value-oriented psychological contract dimensions (*r* ≥ 0.12 ≤ 0.68; small to medium practical effect; *p* ≤ 0.05). Except for the organizational outcome of work-life balance, career adaptability correlated positively and significantly with all the value-orientated psychological contract dimensions (*r* ≥ 0.14 ≤ 0.41; small to medium practical effect; *p* ≤ 0.05). The zero-order correlations were all found to be below the level of concern for multicollinearity (*r* ≥ 0.80). The correlation results prompted an interest to conduct further canonical correlation analyses to assess the ability of agile adaptable attributes (career adaptability and career wellbeing) to predict the value-orientated psychological contract.

**Table 4:** Descriptive statistics and bi-variate correlations.

	Variable	α	CR	Mean (SD)	1	2	3	4	5	6	7	8	9	10	11	12	13	14	15	16	17
1	Positive affect	0.91	0.93	5.12 (1.24)	–																
2	Network/social support	0.82	0.88	5.07 (1.21)	0.66***	–															
3	Meaningfulness	0.89	0.91	5.84 (1.07)	0.70***	0.55***	–														
4	Concern	0.86	0.87	3.95 (0.74)	0.36***	0.36***	0.32***	–													
5	Control	0.79	0.81	3.92 (0.70)	0.30***	0.28***	0.32***	0.52***	–												
6	Curiosity	0.85	0.87	3.95 (0.69)	0.26***	0.32***	0.34***	0.59***	0.51***	–											
7	Confidence	0.89	0.90	4.16 (0.66)	0.27***	0.24***	0.35***	0.49***	0.57***	0.60***	–										
8	Employee’s primary inputs	0.84	0.87	4.45 (0.63)	0.25***	0.20	0.25***	0.35***	0.27***	0.35***	0.41***	–									
9	Employee’s secondary inputs	0.89	0.93	4.20 (0.79)	0.32***	0.23***	0.39***	0.24***	0.26***	0.30***	0.30***	0.62***	–								
10	Organizational outcome: Career development opportunities	0.88	0.88	4.14 (0.89)	0.15**	0.11	0.11	0.25***	0.14*	0.20***	0.15**	0.21***	0.23***	–							
11	Organizational outcome: Organizational culture	0.71	0.77	4.08 (0.85)	0.33 ***	0.29***	0.30***	0.34***	0.27***	0.40***	0.25***	0.30***	0.44***	0.39***	–						
12	Organizational outcome: Relationships	0.85	0.86	4.45 (0.63)	0.12 *	0.09*	0.16**	0.20***	0.19**	0.16**	0.16**	0.21***	0.22***	0.43***	0.49***	–					
13	Organizational outcome: Job characteristics	0.80	0.82	4.43 (0.60)	0.17 **	0.19***	0.29***	0.23***	0.27***	0.35***	0.33***	0.30***	0.30***	0.35***	0.57***	0.56***	–				
14	Organizational outcome: Rewards	0.83	0.84	4.23 (0.81)	0.09	0.06	0.04	0.21***	0.16*	0.20***	0.14*	0.17**	0.11	0.50***	0.37***	0.37***	0.29***	–			
15	Organizational outcome: Work-life balance	0.87	0.88	4.08 (0.82)	0.18**	0.22***	0.13*	0.09	0.09	0.15**	0.10	0.17**	0.07	0.16**	0.29***	0.30***	0.39***	0.17**	–		
16	Psychological contract fulfilment	0.92	0.93	3.45 (1.00)	0.68***	0.54***	0.48***	0.20***	0.23***	0.18**	0.16**	0.23***	0.34***	0.04	0.26***	0.04	0.13 *	0.06	0.18**	–	
17	Employee inputs delivered	0.87	0.89	4.17 (0.77)	0.27***	0.28***	0.36***	0.22***	0.27***	0.28***	0.34***	0.48***	0.45***	0.18**	0.44***	0.33***	0.38***	0.07	0.18***	0.27***	–

***p ≤ 0.001; **p ≤ 0.01; *p ≤ 0.05.

### Canonical correlations

From the canonical correlation analysis, seven canonical functions for the model were derived. Wilk’s lambda was used to assess the null hypothesis that the canonical correlation coefficients for all functions would be zero. For this model, three (out of the seven) canonical functions were found to be significant (*p* < 0.01). A Wilk’s lambda (*λ*) of 0.283, *F*(63, 1,549,29) = 6,174, and *p* < 0.001 were obtained, indicating that the full canonical model was statistically significant across the seven functions. These results indicate that there is a significant and positive association between agile adaptable canonical variates and the psychological contract. The magnitude of the relationship (yielded by 1 – *λ*: 1–0.28) was 0.72 (large practical effect; Fp < 0.001), which indicates that the full model explained a considerable percentage (72%) of the variance shared between the two sets of variables. Refer to Table 5.

**Table 5:** Standardized canonical correlation analysis.

Variate	Variables	Standardized canonical coefficients (canonical weights)	Structure coefficient (canonical loading, Rc)	Canonical cross-loading (Rc)	Squared multiple correlations (Rc*^2^)*
Agile adaptable attributes (canonical variate)	Concern	0.08	−0.42	−0.30	0.09
	Control	−0.09	−0.41	−0.30	0.09
	Curiosity	−0.14	−0.42	−0.30	0.09
	Confidence	−0.01	−0.36	−0.26	0.07
	Positive affect	−0.77	−0.97	−0.70	0.49
	Meaningfulness	−0.01	–0.73	–0.53	0.28
	Networking/social support	–0.25	–0.79	–0.57	0.33
Psychological contract (canonical variate)	Employee’s primary inputs	–0.08	–0.39	–0.28	0.08
	Employee’s secondary inputs	0.01	–0.48	–0.35	0.12
	Employee’s inputs delivered	–0.09	–0.45	-0.32	0.10
	Organizational outcome: Relationships	0.04	–0.19	–0.14	0.02
	Organizational outcome: Career development opportunities	–0.10	–0.22	–0.16	0.03
	Organizational outcome: Rewards	0.05	–0.15	–0.11	0.01
	Organizational outcome: Work-life balance	–0.06	–0.30	–0.22	0.05
	Organizational outcome: Job characteristics	0.00	–0.32	–0.23	0.05
	Organizational outcome: Organizational culture	–0.24	–0.52	–0.38	0.14
	Organizational outcome: Psychological contract fulfilment	–0.83	–0.94	–0.68	0.47
*Overall model fit measure (function 1):*					
*Chi-square (70) = 5.692; p < 0.001; r = 0.726*					

The canonical correlation for the first function was 0.73, and this function contributed 53.1% (Rc^2^; large practical effect) of the explained variance relative to the seven functions. The second and third canonical function (Rc = 0.49 and Rc = 0.37, respectively) explained only a further 24.2% and 13.5% of the variance shared between the two canonical variate sets. The first function was therefore considered practically appropriate for understanding the links between the two sets of variables. From Table 5, it is evident that career wellbeing variates (positive affect, career meaningfulness, and networking/social support) had the most significant predictive ability with regard to the psychological contract variable (Rc^2^ ≤ 0.28 ≥ 0.49).

### Structural equation model

Using the canonical correlation results and to further test the overall structural model fit, structural equation modeling was performed. The fit statistics showed that the tested model fits the data satisfactorily and that the model can be accepted: Chi (19) = 3.06, RMSEA = 0.070, SRMR = 0.21, CFI = 0.81, TLI = 0.80. The goodness-of-fit statistics confirmed the agile adaptable attribute as a significant predictor of the value-oriented psychological contract construct (0.63; *p* = 0.000).

**Figure 1:**
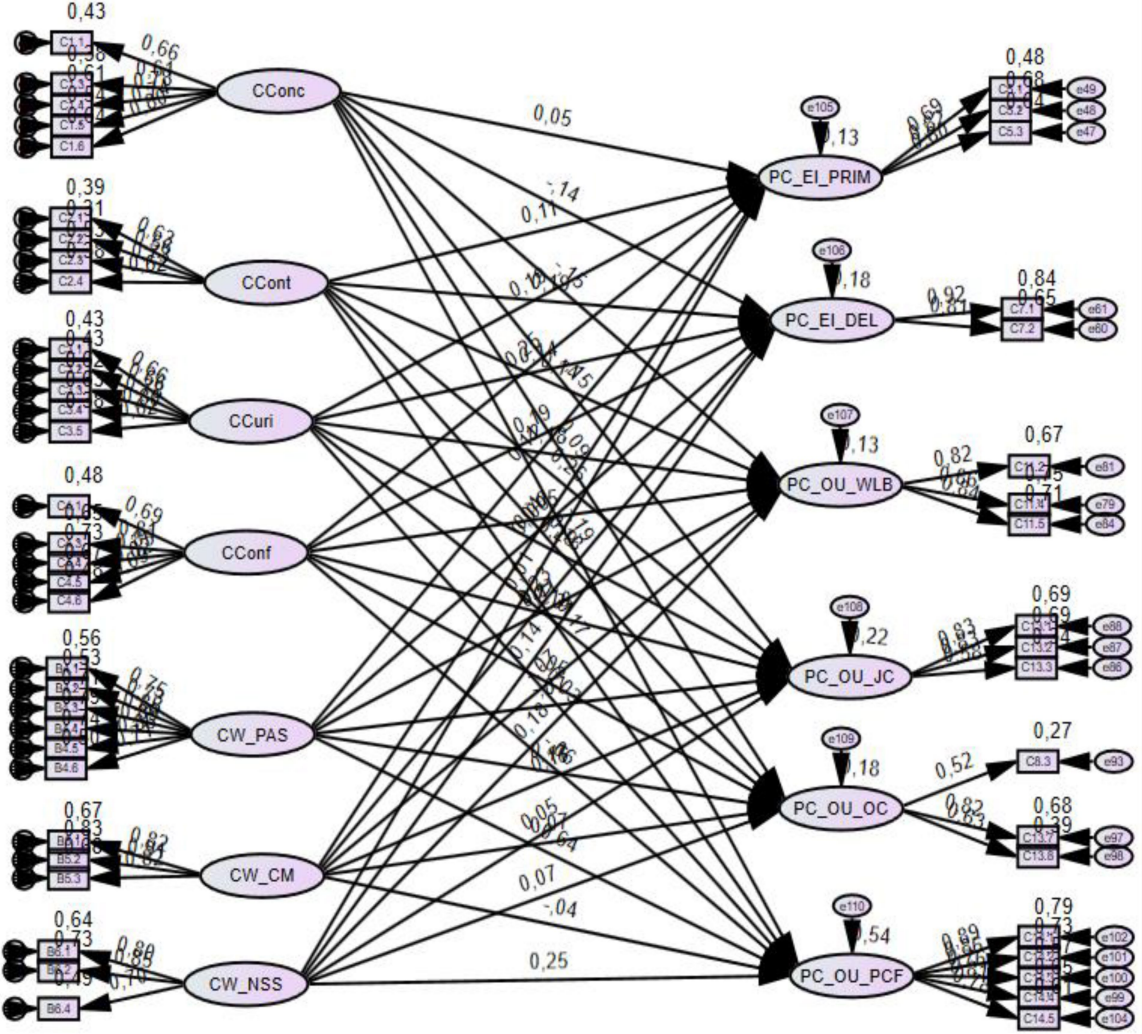
Model fit summary.

Based on the goodness-of-fit model, Figure 1 illustrates the psychological fortitude model recommended for digitally orientated working adults in the digital age.

## Discussion

For organizations to perform optimally, thrive, maintain and increase competitive advantages and adapt to the continuously changing digital world of work, they need employees with considerable psychological fortitude. Organizations should determine what elements contribute toward the psychological fortitude of employees. The central hypothesis of this study was that the agile adaptable construct variables (career wellbeing and career adaptability) would have a direct relationship with the perceived value-orientated psychological contract. Based on the empirical results of this study, a psychological fortitude model for digitally orientated working adults in the digital age is recommended.

The findings of this study seem to reveal close theoretical and empirical associations between career adaptability and career wellbeing (as agile adaptable variables) and the perceived value-orientated psychological contract. The aim of this study was to provide a psychological fortitude model that organizations and career practitioners can use as a basis to enhance employees’ psychological fortitude, and for further career research and career practices.

When individuals are satisfied with the primary (such as task requirements) and secondary (such as working attitude) inputs they deliver to the organization, they will show greater career adaptability (concern, control, curiosity and confidence) and career wellbeing (positive affect, social support/networking, and career meaningfulness). This confirms the theoretical assumption made that individuals with high subjective career satisfaction related to the perception of the value of their contribution to the organization and objectives of the organization experience greater wellbeing (Bester and Bester, 2021) and display greater adaptability (Potgieter, 2021).

Individuals who experience greater career wellbeing are typically satisfied with what they receive back from the organization in return for the services they deliver (although the results showed that career wellbeing did not significantly correlate with the organizational outcome of rewards). This finding contradicts the finding of Chinyamurindi (2019), who found that remuneration influences the wellbeing of employees. Othman et al. (2019) also found that rewards such as salary and promotion have a significant influence on the career satisfaction of employees. Refining the research is therefore necessary in order to differentiate between the types of rewards (intrinsic or extrinsic) that may influence the wellbeing of individuals.

It was found that highly adaptable employees are satisfied with the organizational outcomes received from the organization in return for their services (although no correlation was found between career adaptability and work-life balance). It is thus evident from the empirical results that the agile adaptable construct variables significantly and positively influence the perceived value-orientated psychological construct variable.

**Figure 2:**
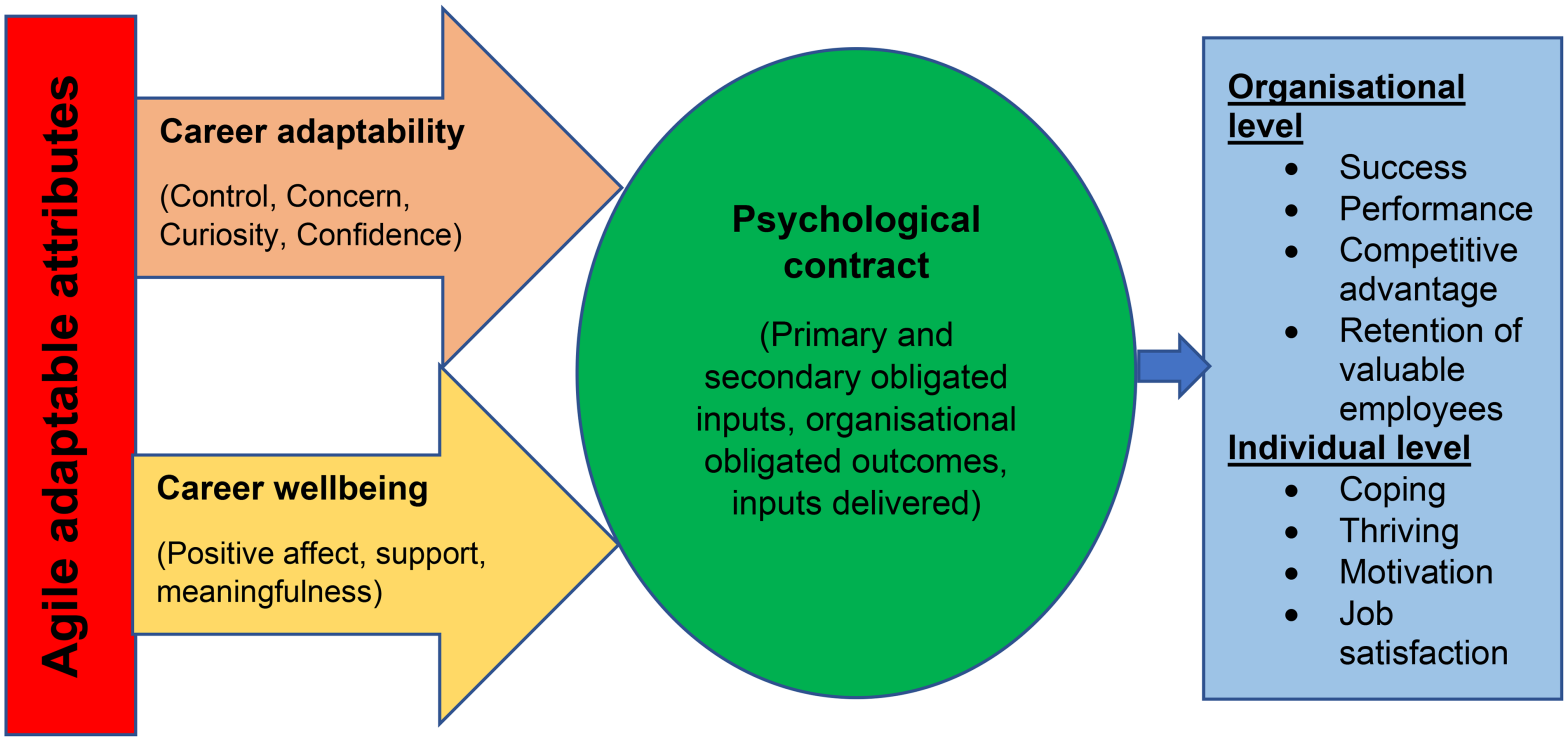
Psychological fortitude model for digitally mindset working adults.

Figure 2 provides an overview of the empirically manifested psychological fortitude model. This profile may be implemented when developing career management practices and interventions for enhancing the psychological fortitude of working adults in the digital era. Enhanced psychological fortitude contribute to success on both an individual and organizational level.

Individuals who do not experience positive relationships at work (positive perceptions about their psychological contract), may break their psychological contract in the career space and search for more meaningful and satisfying, value-orientated work somewhere else. For employees to have a sound psychological contract and thus great psychological fortitude, organizations and career practitioners should implement interventions to enhance the personal enablers of agile adaptable variables. Interventions should include strategies for enhancing career wellbeing and career adaptability. Such interventions may include creating a conducive environment and positive culture to enhance positive career affect, creating platforms to engage in supportive relationships with colleagues, as well as providing meaningful work and job tasks to employees. Interventions should further include strategies to enhance employees’ perception of their career control and their confidence in their career prospects and future, and to awaken career curiosity and career concern. Should employees thus acquire agile adaptable attributes (that is great career adaptability and career wellbeing), they will have positive perceptions about their value-orientated psychological contract with their employer. Good appraisals about the value-orientated psychological contract will create and enhance employees’ psychological fortitude to survive and thrive within the digital career space.

## Conclusion

The results of this study provide empirical evidence that career adaptability and career wellbeing are important attributes in understanding and enhancing the value-oriented psychological contract. The study emphasizes the need to understand the effect of the intrapersonal agile adaptable capabilities/value-oriented psychological contract link. Such understanding may result in and contribute to the psychological fortitude of digitally-oriented working adults. Our anticipation is that the study will inspire future research, especially on the influence of psychological attributes on the value-oriented psychological contract in the digital workspace and new-normal working context.

## Limitations

The study used a cross-sectional research design in collecting the data. Future studies could adopt a longitudinal research design to investigate agile adaptable attributes in relation to the value-oriented psychological contract. In addition, this study was limited to the financial services and human resource management industry. Replication studies should be conducted across a wider industry range and larger samples should be used.

## Data availability statement

The raw data supporting the conclusions of this article will be made available by the authors, without undue reservation.

## Ethics statement

The studies involving human participants were reviewed and approved by University of South Africa (ERC Ref#: 2020_CEMS/IOP_014). The patients/participants provided their written informed consent to participate in this study.

## Author contributions

All authors listed have made a substantial, direct, and intellectual contribution to the work and approved it for publication.

## Conflict of interest

The authors declare that the research was conducted in the absence of any commercial or financial relationships that could be construed as a potential conflict of interest.

## Publisher’s note

All claims expressed in this article are solely those of the authors and do not necessarily represent those of their affiliated organizations, or those of the publisher, the editors and the reviewers. Any product that may be evaluated in this article, or claim that may be made by its manufacturer, is not guaranteed or endorsed by the publisher.
